# Synergistic Effects of Bitumen Plasticization and Microwave Treatment on Short-Term Devulcanization of Ground Tire Rubber

**DOI:** 10.3390/polym10111265

**Published:** 2018-11-13

**Authors:** Łukasz Zedler, Marek Klein, Mohammad Reza Saeb, Xavier Colom, Javier Cañavate, Krzysztof Formela

**Affiliations:** 1 Department of Polymer Technology, Faculty of Chemistry, Gdańsk University of Technology, Gabriela Narutowicza 11/12., 80–233 Gdańsk, Poland; lukzedle@student.pg.edu.pl; 2 Institute of Fluid Flow Machinery, Polish Academy of Sciences, Fiszera 14, 80-231 Gdańsk, Poland; marek.klein@imp.gda.pl; 3 Department of Resin and Additives, Institute for Color Science and Technology, 16765-654 Teheran, Iran; saeb-mr@icrc.ac.ir; 4 Department of Chemical Engineering, Universitat Politècnica de Catalunya Barcelona Tech, Carrer de Colom, 1, 08222 Terrassa, Barcelona, Spain; xavier.colom@upc.edu (X.C.); francisco.javier.canavate@upc.edu (J.C.)

**Keywords:** waste tire recycling, bitumen plasticization, microwave treatment, synergistic effects, structure-property relationships

## Abstract

Ground tire rubber (GTR) was mechano-chemically modified with road bitumen 160/220 and subsequently treated using a microwave radiation. The combined impact of bitumen 160/220 content and microwave treatment on short-term devulcanization of GTR was studied by thermal camera, wavelength dispersive X-ray fluorescence spectrometry (WD-XRF), static headspace, and gas chromatography-mass spectrometry (SHS-GC-MS), thermogravimetric analysis combined with Fourier transform infrared spectroscopy (TGA-FTIR), oscillating disc rheometer and static mechanical properties measurements. The obtained results showed that bitumen plasticizer prevents oxidation of GTR during microwave treatment and simultaneously improves processing and thermal stability of obtained reclaimed rubber.

## 1. Introduction

Dynamic development of the automotive industry is responsible for the continuous increase of post-production and post-costumer waste rubber. For example, a compact car contains around 60 kg of rubber, the tires constitute about 70% of the total rubber weight [1]. According to European Tyre Rubber Manufacturers Association statistics, in 2016, the production of tires in the European Union increased to 494 million tons (~25% of global production) with an approximate growth of 1% annually [2]. On the other hand, estimated data indicate that around 1000 million waste tires are discarded worldwide each year, more than 50% of them are directly discarded, landfilled or burned [3]. This presents a serious threat to the natural environment. Therefore, searching for new and pro-ecological reutilization methods of used tires and other waste rubbers is one of the biggest challenges of the 21st century waste management [4].

As mentioned above, nowadays, energy recovery is the most used method, which utilizes waste tires as alternative fuel in cement kilns and power plants [5]. The common acceptance to this solution is related mostly to economic factors because alternative industrial technologies are still in a low degree of development in order to provide competitive environmental-friendly utilization or “up-cycling” of waste tires.

In this context, during the last two decades, many attempts have been focused on laboratory applications of GTR. An option is to include GTR as a filler or modifier in different polymer composites, which were comprehensively reviewed in works of Karger-Kocsis et al. [6], Ramarad et al. [7], and Sienkiewicz et al. [8]. Another interesting approach is the valorization of waste rubber by reclaiming. This process, sometimes called in the literature as devulcanization, is related to the transformation of waste rubber using thermal, mechanical or chemical energy, in order to destroy the three-dimensional network of a cross-linked rubber. Compared to untreated waste rubber, the obtained reclaimed rubber can be easily processed, shaped, and vulcanized [9,10].

Waste rubber reclaiming is usually performed at high temperature, high shear forces, and specific conditions, which are necessary for the scission of cross-linking bonds present in rubber. The reclaiming conditions strongly affect the course of chemical degradation reactions, having an influence on the balance between pursued selective scission of cross-linking bonds and the undesired degradation of main polymeric chains, consequently determining the final properties of obtained reclaimed rubber [11,12,13].

According to recent literature, “green” reclaiming/devulcanization methods applied to GTR constitute a very promising approach to provide alternative utilization of waste rubber. Research studies in this field are usually focused on combining low temperatures and short time of processing. These conditions allow to reduce energy consumption and production costs while simultaneously prevent the emission of hazardous volatile organic compounds during GTR reclaiming [14,15,16]. Moreover, published data also indicates that lower temperature during GTR reclaiming allows the selective scission of cross-linking bonds. Additionally, limited degradation of the main polymeric chains enhances the mechanical properties of the resulting material [17,18]. Under these conditions, the possibility to meet the strict requirements of sustainable development for currently used reclaiming/devulcanization technologies is open, therefore, further research on this area is fully justified.

The main disadvantage of low-temperature reclaiming might be the technological problems related to the processing of GTR [19]. In order to overcome this limitation, the application of plasticizers suitable to increase flexibility or workability of GTR during low-temperature reclaiming has been proposed [20,21]. Another solution is applying a microwave treatment to GTR [22,23,24], which allows devulcanization of GTR in a short-time, affecting its structure, morphology, processing, and consequently its final performance properties. However, according to the our best knowledge, the studies regarding the combined effects of GTR bitumen plasticization and microwave treatment were not published so far.

In this work, GTR was mechano-chemically modified with road bitumen 160/220, which was used as reactive plasticizer. The modification was performed at ambient temperature using a two-roll mill, which allowed the generation of suitable shear forces on GTR and reduce energy consumption during the process. The obtained products were subsequently treated by microwave radiation. For better understanding of synergistic effects of bitumen plasticization and microwave treatment on short-term devulcanization of ground tire rubber (GTR), the impact of bitumen content (in the range of: 0–2.5 phr) on structure-properties of resulting materials was determined.

## 2. Materials and Methods

### 2.1. Materials

**Figure 1:**
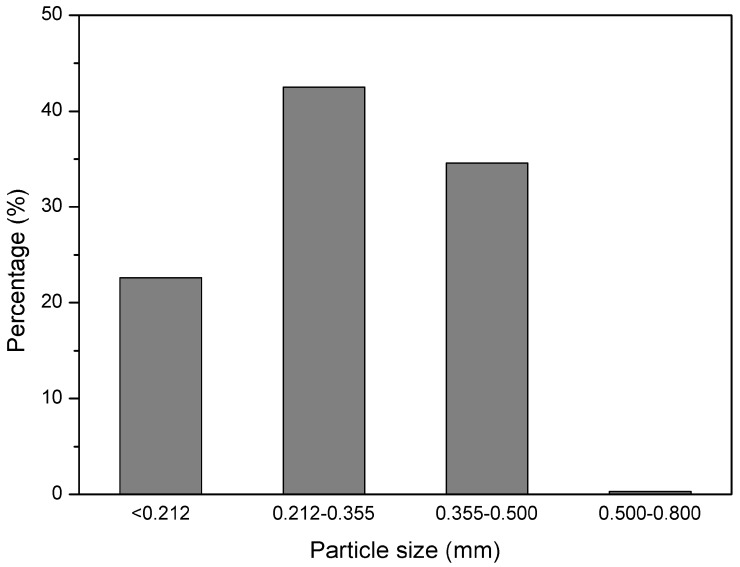
Particle size distribution of GTR.

GTR with particles size below 0.5 mm was received from Grupa Recykl S.A. (Śrem, Poland). GTR was obtained by ambient grinding of used tires (mix of passenger car tires and truck tires). The particle size distribution of used GTR is presented in Figure 1. Road bitumen 160/220 with penetration at 25 °C: 170–210 (1/10 mm) and softening point: 35–43 °C was received from Lotos Asfalt Sp. z o.o. (Gdańsk, Poland). 

### 2.2. Sample Preparation

#### 2.2.1. GTR Modification and Microwave Treatment

GTR was processed at ambient temperature by means of two-roll mill model 14201/P2 from Buzuluk (Czech Republic). In order to improve the GTR processing during mechano-chemical devulcanization, GTR was modified with a variable content of bitumen (in the range of: 0–2.5 phr) as a reactive plasticizer. Low-temperature mechano-chemical treatment of GTR was performed using a high shear forces (small gap) for 10 min according to the procedure described in our patent application [25]. The following two-roll mill settings were used: ambient temperature, friction equaled 1.08 and the gap width varied between 0.2 and 3 mm. The mechano-chemical treatment of GTR allows the formation of sheets with constant thickness (3 mm). The obtained sheets were cut into circular samples with constant weight (65 g), which were put directly on turn-table and subsequently treated by microwave radiation using a domestic microwave R270W from Sharp (Osaka, Japan). The power of the magnetron oven was set up to 800 W. Preliminary investigations showed that reference sample (GTR without bitumen) cannot be treated with microwaves under these conditions for a period longer than 120 s, otherwise the material burnt. The ignition can be related to the evaporation of low molecular compounds and their inflammation. The microwave specific energy *E* (Wh/kg) during microwave treatment can be estimated by Equation (1):

(1) $$\;E=\frac{P\times t}{m}$$

where: *P*—microwave power (W), *t*—radiation time (h), and *m*—weight of sample (kg).

Based on preliminary investigation results, for all studied samples microwave power (*P* = 800 W) and radiation time (*t* = 120 s) were assumed to be constant. These settings resulted in a received microwave specific energy equal to 410 Wh/kg. This value corresponds with data published by Seghar et al. [26], who used 440 Wh/kg as maximal microwave energy to perform controlled devulcanization of GTR. 

The samples were coded as GTR+YB-MW, where Y means bitumen 160/220 (B) content. For example, GTR+2.5B-MW is a sample of GTR modified with 2.5 phr of bitumen 160/220, which was subsequently microwave-treated. GTR without prior bitumen modification and processed in the same conditions was used as a reference sample and coded as GTR-MW.

These samples were studied by the thermal camera, wavelength dispersive X-ray fluorescence spectrometry (WD-XRF), static headspace and gas chromatography-mass spectrometry (SHS-GC-MS) and thermogravimetric analysis combined with Fourier transform infrared spectroscopy (TGA-FTIR). In order to evaluate the mechanical properties of the final product, the vulcanization process was carried out.

#### 2.2.2. Vulcanization of Reclaimed GTR

In order to determine the combined impact of bitumen plasticization and microwave treatment on the curing behavior and performance properties of GTR after short-term microwave-induced devulcanization, the obtained samples were mixed with a sulfur curing system using two-roll mill from Buzuluk (Komárov, Czech Republic).

For all samples the same curing system was used. The composition in parts per hundred of rubber (phr) was: stearic acid 1.0; zinc oxide 2.5; TBBS (*N*-*tert*-butyl-2-benzothiazole sulfenamide) 0.35; sulfur 1.5.

The samples were shaped in sheets with 2 mm thickness and then cured in a electric heated press at 150 °C under a pressure of 4.9 MPa for the optimum vulcanization time (*t*_90_) determined by an oscillating disc rheometer according to the ISO 3417 standard.

### 2.3. Measurements

Temperature distribution into reclaimed GTR after microwaves treatment was measured using a infrared thermal imaging camera model InfRec R300SR from NEC Avio Infrared Technologies (Tokyo, Japan). The characteristics of InfRec R300SR is presented in Table 1.

Weight loss after microwave treatment of GTR was determined as the mass difference of samples before (W_1_) and after treatment (W_2_), according to Equation (2):

(2) $$\;\mathrm{Weight}\;\mathrm{loss}\;\mathrm{after}\;\mathrm{microwave}\;\mathrm{treatment}=\frac{W_{1}-W_{2}}{W_{1}}\times 100\%$$

Temperature and weight loss after MW treatment of GTR were measured at least three times per sample and the obtained results are presented as median values.

The content of the elements in microwave treated GTR was determined by wavelength dispersive X-ray fluorescence spectrometry (WD-XRF) using a spectrometer S8 Tiger 1KW from Bruker (Billerica, MA, USA). Examined samples were put into dishes for powder studies on Prolen^®^ foil with thickness 4 µm. Measurements were performed in a helium atmosphere.

Volatile organic compounds (VOCs) emitted from reclaimed GTR were determined using static headspace and gas chromatography-mass spectrometry (SHS-GC-MS). Measurements were performed using a Shimadzu GC2010 PLUS GC-MS (Shimadzu Corporation, Kioto, Japan) equipped with a split/splitless inlet. The GC-MS system was equipped with an AOC5000 Headspace Auto-Sampler. During analysis, the vial was transported by the injection unit from the tray to the agitator; when the sample achieved the equilibrium, the headspace sample of 2.5 mL volume was drawn from the vial and injected into the GC injector. The sampled vial was then returned by the injection unit to the tray. Conditions and parameters of SHS-GC-MS analysis are summarized in Table 2.

The thermal analysis of GTR after microwaves treatment was performed using the simultaneous TGA/DSC model Q600 from TA Instruments (New Castle, DE, USA). Samples of reclaimed GTR weighing approx. 10 mg were placed in a corundum dish. The study was conducted in an inert gas atmosphere—nitrogen (flow rate 100 mL/min) in the range from 25 to 800 °C with a temperature increase rate of 20 °C/min. Volatile products from thermal degradation of studied samples were also evaluated using a Fourier transform infrared spectroscopy (FTIR). During TGA/DSC measurements volatile degradation products were directed (using heated transfer line with temperature 220 °C) to Nicolet iS10 spectrometer from Thermo Scientific (Waltham, MA, USA). The presented solution allows “on-line” characteristics of volatile products during TGA/DSC measurements. The timing offset of FTIR spectra comparing to TGA curves is related with a volume of thermogravimetric apparatus chamber.

The curing process of reclaimed GTR samples was investigated at 150 °C, using a Monsanto R100S (Monsanto Company, St. Louis, MO, USA) rheometer with an oscillating rotor according to ISO 3417. Oscillation angle was 3° and torque range 0–100 dNm. Cure rate index values were calculated in accordance with the Equation (3):

(3) $$\;\mathrm{CRI}=\frac{100}{t_{90}-t_{2}}$$

where: *t*_90_—optimum vulcanization time, min; *t*_2_—scorch time, min.

In order to determine the aging resistance of studied vulcanizates at elevated temperatures, R_300_ parameter was determined. R_300_ defines the percentage reversion degree after a period of 300 s calculated from the time of reaching maximum torque (M*_H_*). R_300_ was calculated in accordance with Equation (4):

(4) $$\;R_{300}=\frac{M_{H}-M_{300s}}{M_{H}}\times 100\%$$

where: M*_H_*—maximum torque; M_300*s*_—torque 300 s after maximum torque.

The tensile strength, elongation at break and modulus at 100% of elongation (M_100_) were estimated in accordance with ISO 37. Tensile tests were performed on the Zwick Z020 machine (Zwick Roell Group, Ulm, Germany) at a constant speed of 500 mm/min. Direct extension measurements were conducted periodically using an extensometer with sensor arms. The reported results stem from five measurements for each sample. Shore hardness type A was estimated using Zwick 3130 durometer (Zwick Roell Group, Ulm, Germany) in accordance with ISO 7619-1. 

## 3. Results and Discussion

### 3.1. Temperature Distribution and Weight Loss of GTR after MW Treatment

**Figure 2:**
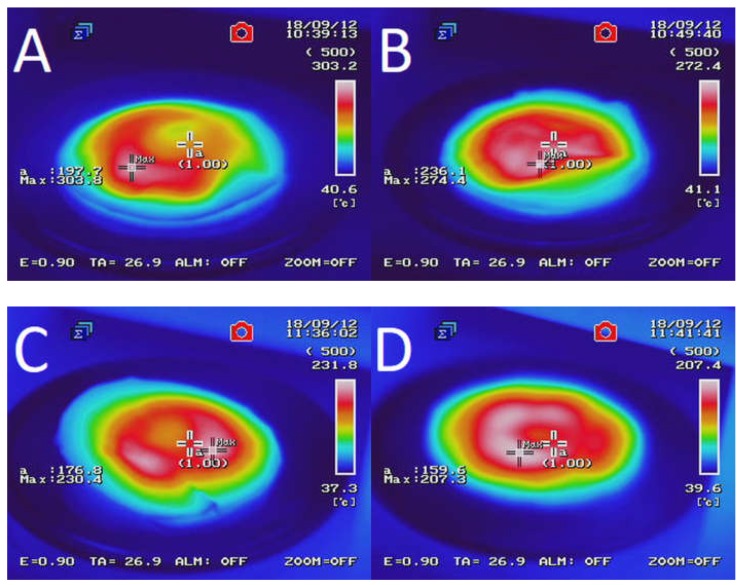

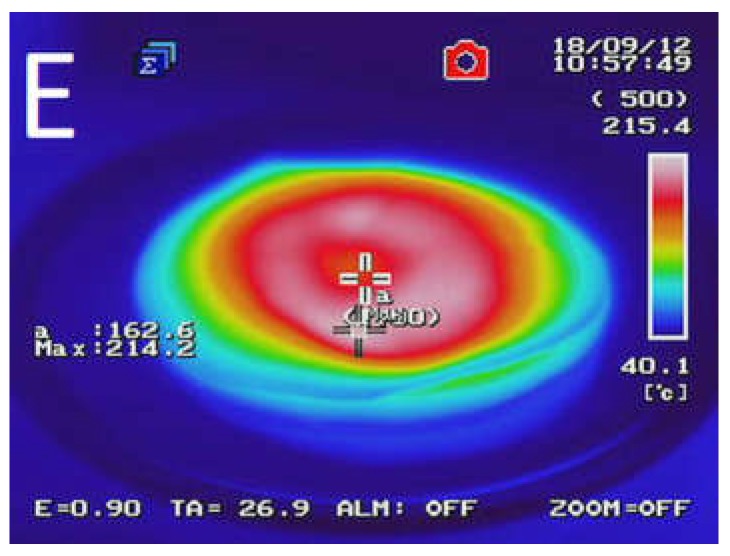
Temperature distribution measured with a thermal camera for sample: (**A**) GTR-MW; (**B**) GTR+0.25B-MW; (**C**) GTR+0.5B-MW; (**D**) GTR+1.0B-MW; and (**E**) GTR+2.5B-MW.

Figure 2 presents the temperature distribution into GTR after microwave treatment as a function of bitumen 160/220 content. The results determined by using an InfRec R300SR infrared thermal camera and weight loss of samples after MW treatment are summarized in Table 3. It was observed that maximal and average temperatures of reclaimed GTR gradually decreasing with higher bitumen content. Comparing to sample GTR-MW, application of 2.5 phr of bitumen 160/220 in sample GTR+2.5B-MW caused a decrease of maximal temperature and the average temperature by 89.6 °C and 35.1 °C, respectively. This phenomenon is related to lower carbon black content in studied materials due to partial substitution of GTR by bitumen, which affects the efficiency of Maxwell-Wagner polarization effect. The results of temperature distribution also indicate that during MW treatment bitumen acts as an insulator and protect GTR from uncontrolled oxidation and ignition, which resulted in lower values of weight loss after MW treatment (for about 62%) than GTR-MW sample (the exception was the sample with the smallest amount of bitumen). Additionally, it was found that using bitumen as a plasticizer has a beneficial impact on temperature distribution into reclaimed GTR compared to the reference sample (GTR-MW).

### 3.2. WD-XRF Analysis of Reclaimed GTR

For a better understanding of this phenomenon, elemental analysis of studied materials was performed using wavelength dispersive X-ray fluorescence spectrometry (WD-XRF) and the obtained results are presented in Table 4.

This non-destructive method allows rapid and quantitative determination of content of silicon (Si), sulfur (S), zinc (Zn), calcium (Ca), magnesium (Mg), aluminum (Al), and iron (Fe) in rubber compounds [27,28]. Miskolczi et al. [29], and recently Liang et al. [30], confirmed that the most intense signal from X-ray fluorescence spectrometry corresponds to zinc, and it is related to its high concentration in GTR. In the studied case the range is 1.22–2.33 wt %. It was noticed that Zn concentration significantly decreased after MW treatment compared to untreated GTR. This could be explained by partial thermal decomposition of zinc stearate (activator used during rubber compounding) present on the vulcanized rubber surface [31], which, according to the literature, occurs at around 250 °C.

Another strong signal detected by WD-XRF is related to sulfur, commonly used as a curing agent in rubber compounds. The results showed that sulfur concentration in studied samples was in the range of: 1.33–2.16 wt %. The lowest value was determined for GTR-MW sample, while the highest was found in case of the sample with 2.5 phr of bitumen (coded as GTR+2.5B-MW). Sulfur is responsible for the formation of cross-linking bonds during vulcanization. Therefore, its content could put some insight on cross-link density of studied samples. Based on this assumption, the sulfur content value indicates that scission of cross-linking sulfide bonds occurs more efficient for GTR-MW sample than for GTR+2.5B-MW sample. The measurements obtained by oscillating disc rheometer discussed later seem to confirm this statement. On the other hand, it should be pointed out that there is no simple correlation between sulfur content and cross-link density [30,32]. Higher sulfur content in the case of GTR samples modified with bitumen could also be explained by sulfur derivatives (such as hydrogen sulfide, carbon disulfide, etc.) present in the bitumen [33].

Silicon content determined by WD-XRF was in the range of 1.72–2.90 wt %, which corresponds with the presence of silica—commonly used as filler incorporated to tires in order to decrease their rolling resistance. Moreover, WD-XRF analysis detected Ca—0.38–0.62 wt %, Al—0.07–0.14 wt %, Mg—0.06–0.09 wt %, and Fe—0.04–0.14 wt %. It was found that microwave treatment of GTR did not affect the content of Si, Ca, Al, and Mg, while the concentration of Fe significantly decreased. This could be related to partial iron oxidation supported by microwave treatment. As could be observed, bitumen plasticization of GTR resulted in a higher content of Si, Ca, Al, Mg, Fe in GTR+2.5B-MW sample comparing to GTR-MW sample or untreated GTR. This phenomenon can be explained by possible migration of silica and other inorganic particles into GTR surface due to the higher mobility of polymer chains resulted from combined impact bitumen plasticizer and microwave treatment. Additionally, it should be mentioned that these elements can also be present in bitumen [34].

### 3.3. SHS-GC-MS Analysis of Reclaimed GTR

During further studies, we decided to estimate the impact of bitumen plasticizer content on the emission of volatile organic compounds (VOCs) during MW treatment. It is well known that VOCs have a negative impact on the environment, which might be a serious problem for novel technologies due to environmental regulations. However, assessment of VOCs released from polymers and recycled polymers as a function of variable processing conditions is rather poorly described in the literature [35,36,37]. Volatile organic compounds identified using a SHS-GC-MS method are presented in Table 5. Applied measurement conditions allows for determination of seven compounds: acetone (content in the range of: 1.5–2.5 mg/kg), methacrolein (0.5–0.9 mg/kg), 2-methylfuran (0.6–1.3 mg/kg), methyl vinyl ketone (0.9–1.6 mg/kg), methyl isobutyl ketone (3.9–8.3 mg/kg), cyclohexanone (1.6–2.4 mg/kg), and benzothiazole (6.5–7.9 mg/kg). Regardless of GTR treatment conditions, methyl isobutyl ketone and benzothiazole were determined in the highest concentration. Methyl isobutyl ketone is the main component during synthesis of antiozonant 6PPD—commonly applied in tires. Benzothiazole is the partial structure of vulcanization accelerators used during manufacturing of rubber compounds. Detection of benzothiazole corresponds to the presence of unreacted curing system or scission of sulfide cross-linking bonds presented in GTR.

The presence of ketones in reclaimed GTR indicates its partial oxidation during microwave treatment. Morand et al. [38] proved that methacrolein and methyl vinyl ketone are products formed during oxidation of polyisoprene, while other determined volatile compounds were also detected in natural rubber [39]. This suggests higher oxidation of natural rubber phase than synthetic rubber phase in GTR. Obtained results corresponds with observations described recently by Sousa et al. [40], who performed comprehensive studies of chemical modifications and thermo-oxidative degradation behavior of GTR as a function of the microwave radiation time.

Surprisingly, the highest total content of VOCs was determined for unmodified GTR, which could be related to SHS-GC-MS analysis conditions, because studied samples were pre-heated at 150 °C for 20 min (see Table 2). This could cause additional emission of VOCs from GTR. It should be also pointed out that formed VOCs could act like plasticizers [41], while their interactions with polymeric matrix could affect the results of SHS-GC-MS. Furthermore, it was observed that volatile organic compounds were omitted directly to the environment after MW treatment of GTR, which also affected the total content of VOCs in studied samples. These factors resulted in a lack of simple correlation between bitumen plasticizer content and determined VOC amounts. 

### 3.4. TGA-FTIR Studies of Reclaimed GTR

**Figure 3:**
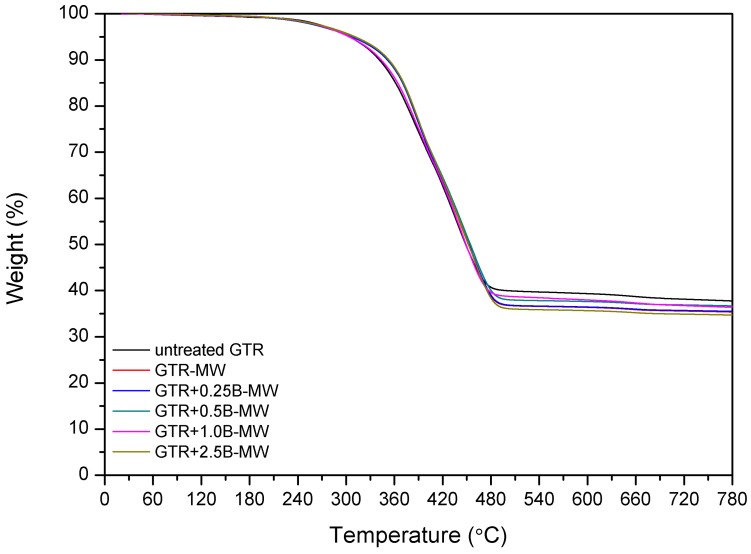

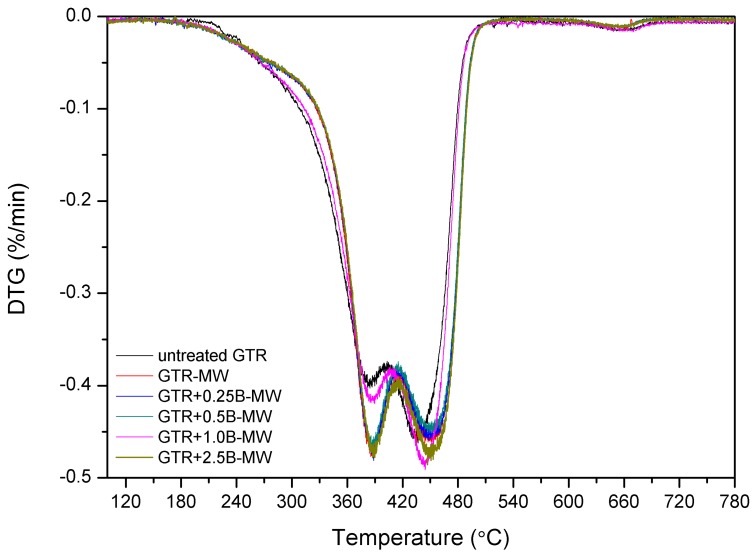
TGA and DTG curves of studied samples.

Kleps et al. [42] and Scuracchio et al. [43] proved that thermogravimetric analysis (TGA) could be a useful analytical tool to estimate the changes in the chemical structure of reclaimed rubber. In this study, we expanded this methodology using TGA conjugated with FTIR, which allowed more detailed characterization of the chemical structure of reclaimed GTR. The curves of thermogravimetric analysis and derivative thermogravimetry (DTG) are presented in Figure 3. 

Thermal analysis was conducted in a nitrogen atmosphere and carried out to complete degradation of the organic components of the sample, in order to determine the stability of the samples and the amount of final residue. The obtained results are summarized in Table 6. 

The data showed that T_−2%_ temperature, which corresponded to the 2% weight loss, was lower for the sample treated with microwaves (GTR-MW) than for untreated GTR. This is due to the reclaiming effect of the microwaves which cause scission of the chains and, subsequently, a higher amount of products susceptible to degradation at lower temperatures. 

However, T_−5%_, T_−10%_, and T_−50%_ temperatures increased after the MW treatment. This phenomenon corresponds to the volatile degradation products generated by the scission of polymeric chains during MW reclaiming process. Partial evaporation of volatile compounds caused that the remaining material becomes more thermally stable after MW treatment.

Char residue results are in line with the usual contents of carbon black in GTR. After MW treatment char residues in GTR are lower, which is related to the elimination of low molecular weight compounds formed during the devulcanization. Garcia et al. [22] proved that carbon black present in GTR could adsorb low molecular volatile compounds generated during thermal degradation (barrier effect), and this phenomenon can improve thermal stability of reclaimed GTR.

The results showed that adding bitumen to the samples has three predominant effects. Firstly, according to the temperature measurements presented before, bitumen decreases the general temperature of the GTR after MW treatment, protecting and preserving its degradation. Secondly, bitumen acts as plasticizer that promotes the diffusion and elimination of low molecular weight compounds by improved mobility through the polymeric chains. Thirdly, factor affecting obtained results is the thermal stability of pure bitumen.

These effects are combined in the studied samples. When adding 0.25 phr bitumen, according to the temperatures determined by the thermal camera, the average temperature achieved was higher than in the case of GTR-MW. This amount of bitumen is insufficient to maintain the sample at a relatively low temperature. The effect of the MW treatment on this sample is similar to the sample without bitumen, therefore scission of chains are comparable. T_−2%_, decreases from 255.8 °C for sample GTR-MW) to 250.5 °C determined for sample GTR+0.25B-MW. This decrease is also related to the plasticizing effect that, as exposed above, favors migration of the low molecular weight degradation products and causes a higher weight loss at lower temperatures. The difference between these two samples at T_−5%_, T_−10%_, and T_−50%_ is not as large as in the case of T_−2%_. Once the low molecular compounds evaporate, the removal of the remaining components is not so favored by the plasticization. The progressive addition of bitumen to the GTR samples tend to increase the T_−2%_, T_−5%_, T_−10%_, and T_−50%_. For example, sample GTR+0.25B-MW has a T_−2%_ of 250.5 °C while the GTR+2.5B-MW sample presents a T_−2%_ of 254.7 °C. The increment of temperatures depends on the amount of bitumen, reaching a maximum when adding 2.5 phr. 

According to the measurements performed with the thermal camera (see Table 3), the GTR+2.5B-MW sample achieved lower temperature after MW treatment than GTR+0.25B-MW sample or reference sample (GTR without bitumen). In this case, the bitumen acted as a protective agent (insulator) that preserves from degradation and consequently decreases the amount of volatile products emitted from the samples. 

The third of the described effects is more evident in the case of GTR+2.5B-MW sample containing 2.5 phr of bitumen and predictably for samples with higher content of bitumen plasticizer. This effect is related to thermal degradation of bitumen. This plasticizer contains low molecular weight components that evaporate and degrade below 300 °C, and also some stable molecules that degrade over 400 °C. Those two facts are the cause of the shift of the decomposition temperatures (T_−10%_ and T_−50%_) of the GTR+2.5B-MW sample towards higher levels when compared to the GTR-MW sample. 

**Figure 4:**
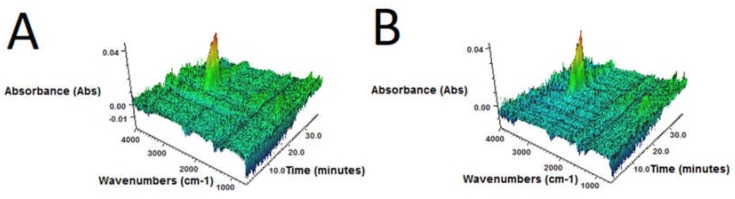

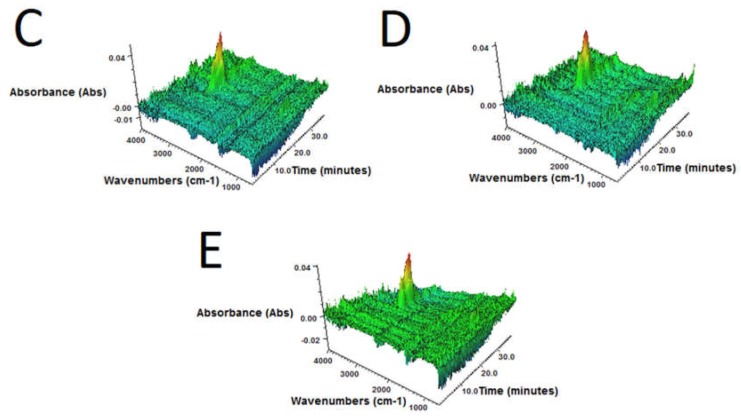
3D FTIR plots determined for volatile products emitted during thermal decomposition of sample: (**A**) GTR-MW; (**B**) GTR+0.25B-MW; (**C**) GTR+0.5B-MW; (**D**) GTR+1.0B-MW; and (**E**) GTR+2.5B-MW.

Figure 4 shows DTG curves with two peaks corresponding to the two main components of GTR. The first one is related to the maximum rate of thermal degradation of NR around 390 °C and the second to styrene-butadiene rubber around 450 °C [44]. The relative intensity of the DTG peaks suggests that the impact of the MW treatment on the samples is different in the NR and SBR domains when bitumen is included. The relative difference of height of the peaks corresponding to NR and SBR is substantial in the case of the sample GTR-MW (without bitumen) while the difference in the relative height of the peaks of the sample GTR+2.5B-MW is smaller. Moreover, it was observed that a higher content of bitumen shifted T_max2_ towards higher temperatures, while T_max1_ values for GTR samples treated by MW were similar. This suggests that SBR domains are more preserved when adding bitumen, that would indicate that the distribution of bitumen on the GTR shows preference for SBR while the NR, less encapsulated by the bitumen is more affected by the MW treatment. This observation corresponds with the results of SHS-GC-MS, which confirms the formulation of volatile organic compounds related to natural rubber/polyisoprene decomposition.

3D FTIR spectra of volatile degradation products emitted during thermal decomposition of samples are present in Figure 4. 

It was found that, regardless of bitumen content, for all samples 3D FTIR spectra were similar. The strongest absorbance bands, situated in the 2800–3000 cm^–1^ region were observed. These signals are attributed to the symmetric and asymmetric stretching vibrations of C–H bonds in CH_2_ groups present in gaseous degradation products of GTR. This indicates that during slow pyrolysis (TGA in inert atmosphere), the main emitted products are aliphatic hydrocarbons, what corresponds with the literature data [45,46].

### 3.5. Curing Characteristics of Reclaimed GTR

The effect of bitumen plasticizer content on curing characteristics of microwave treated GTR is shown in Table 7. It was found that samples GTR-MW and GTR+0.25B-MW were characterized by the lowest minimal torque (M_L_) value, 16.9 dNm, and 17.9 dNm, respectively. These results confirm that microwave treament of GTR enhances processing (M_L_ is strongly correlated with rubber compound viscosity) of the obtained reclaimed rubber. This is due to the high temperatures generated in these samples by the microwave treatment (maximal temperature respectively: 303.8 °C and 274.4 °C, Table 3), which induce scission of cross-linking bonds and cause partial degradation of the polymer main chains. The addition of 0.5 phr bitumen into GTR (sample GTR+0.5B-MW) resulted in a significant increase of M_L_, around 40% compared to sample GTR+0.25B-MW. On the other hand, the application of higher bitumen content cause decrease of M_L_, which confirms that the plasticization effect of GTR by bitumen occurs.

**Figure 5:**
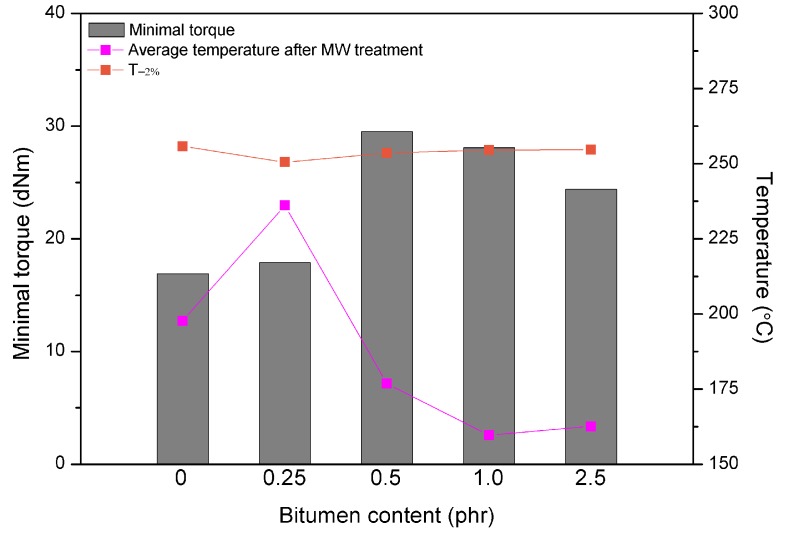
The relationship between minimal torque, average temperature after MW treatment of GTR and T_−2%_ determined by TGA as function of bitumen plasticizer content.

For a better understanding of the synergistic effects of bitumen plasticization and microwave treatment on short-term devulcanization of GTR the relationship between minimal torque, the average temperature of GTR after MW treatment and T_−__2%_ determined by TGA as a function of bitumen content is presented in Figure 5. It could be noticed, that higher content of bitumen (above 0.25 phr) resulted in a decrease of the minimal torque and the average temperature of GTR, while T_−__2%_ parameter after MW treatment increased. These combined results, which are directly related, indicate that at the higher content of bitumen the plasticization effect of GTR is more prevailing than the consequences of the microwave treatment. In this case, bitumen acting as plasticizer preserve oxidation of GTR during the microwave radiation and simultaneously improves processing of reclaimed GTR. 

Maximal torque corresponds with stiffness and shear modulus of vulcanized samples and torque increment (ΔM) is correlated with their cross-link density. The obtained results showed that samples with higher bitumen content have higher stiffness, which confirms that for these samples the microwave treatment had less influence than for sample GTR+0.25B-MW characterized by very a low content of bitumen. On the other hand, scorch time (*t*_2_), optimal cure time (*t*_90_), cure rate index (CRI), reversion degree (R_300_) and torque increment (ΔM) of all studied samples were rather similar, some slight differences could be explained by the complex composition of GTR produced from waste tires.

### 3.6. Static Mechanical Properties of Reclaimed GTR 

Static mechanical properties of vulcanized reclaimed GTR are summarized in Table 8. 

As could be observed, all studied samples showed similar tensile properties, tensile strength (TS_b_) in the range of: 5.2–6.1 MPa, elongation at break (E_b_) 109–139%, modulus at 100% elongation (M_100_) 4.0–4.7 MPa and hardness (H) 63–67° Sh A. This denotes a lack of simple correlation between bitumen plasticization effect and tensile properties of reclaimed GTR. The microwave treatment and bitumen plasticization of GTR result in partial devulcanization and degradation. Then, the obtained reclaimed GTR should be considered as a heterophase composite rather than a homopolymer matrix. Reclaimed GTR is formed by a gel fraction (cross-linked GTR particles that remain after the treatments) and sol fraction (the sum of the devulcanized, degraded and plasticized rubber phase). The formation of a network between cross-linked GTR particles and these different elastomeric chains present in the samples, could explain the slight differences in tensile properties observed for reclaimed GTR. Similar observations have been reported for polyethylene cross-linked waste [47,48]. In order to confirm these assumptions for GTR, tensile properties of obtained reclaimed GTR were compared with other reclaimed rubbers prepared by different methods, as presented in Table 9.

The results from literature showed that reclaimed GTR tensile strength presents a variation of 3.2–8.4 MPa, and elongation at break values are situated in a range comprised in 109–202%. The tensile properties of the studied materials correspond with these values. However, it should be mentioned that apart of the reclaiming method and GTR characteristics (particle size, composition), some other variables related to curing conditions (e.g., curing system, vulcanization settings) have a strong influence on the final performance properties of reclaimed rubber. These considerations have been highlighted in our previous work [54].

## 4. Conclusions

The synergistic effects of bitumen plasticization and microwave treatment on short-term devulcanization of GTR and structure-properties of obtained reclaimed GTR was investigated. The obtained results confirmed that bitumen plasticization of GTR affects the efficiency of microwave treatment and consequently short-term devulcanization course. It was found that during MW treatment bitumen acts as an insulator, which protects GTR from uncontrolled oxidation and ignition. Additionally, bitumen as plasticizer has a beneficial impact on temperature distribution after MW treatment of GTR and improved processing of obtained reclaimed GTR. Furthermore, in the studied conditions, the impact of bitumen plasticizer on performance properties of reclaimed GTR was negligible.

## Figures and Tables

**Table 1:** Characteristics of InfRec R300SR infrared thermal imaging camera.

Item	Specification
Detector	Uncooled focal plane array (microbolometer)
Number of pixels	320 (H) × 240 (V) (Three edge lines of the screen is out of the specs)
Measuring range	0 to 500 °C
Spectral range	8 to 14 µ
Noise equivalent temperature difference (NETD)	0.3 °C (at 30 °C)
Temperature indicating accuracy	At ambient temperature over −15 to 50 °C: ±2 °C or ±2% of readings, whichever is greater
Instantaneous field of view	1.21 mrad
Field of view	22° (horizontal) × 17° (vertical), accuracy: ±10%
Frame time	60 Hz
A/D resolution	14 bit

**Table 2:** The conditions and parameters of SHS-GC-MS analysis of reclaimed GTR.

**Instrumental GC Analysis Parameters**				
	Carrier Gas		Helium	
Inlet	Injector mode		Split	
	Split Ratio		10	
	Flow		2.0 mL/min	
	Temperature		220 °C	
Oven	Column		DB-624 60m	
	Temp. (°C)	Rate (°C/min)	Final temp. (°C)	Hold (min)
	35	0	35	4.5
	35	10	200	-
	200	30	290	5
Mass Spectrometer	Solvent cut time		5 min	
	Ion source temp.		220 °C	
	Interface temp.		245 °C	
	Scan range		35–350 *m/z*	
**Headspace Parameters**				
	Incubator temp		150 °C	
	Syringe temp		160 °C	
	Incubation time		20 min	
	Injected volume		2.5 µL	

**Table 3:** Characterization of studied samples after MW treatment.

Item	Methodology	GTR-MW	GTR+0.25B-MW	GTR+0.5B-MW	GTR+1.0B-MW	GTR+2.5B-MW
Weight loss after MW treatment (%)	Analytical electronic balance	0.59	0.53	0.38	0.36	0.37
Average temperature of GTR after MW treatment (°C)	Thermal camera	197.7	236.1	176.8	159.6	162.6
Maximal temperature of GTR after MW treatment (°C)		303.8	274.4	230.4	207.3	214.2

**Table 4:** WD-XRF analysis for studied samples.

Element (wt %)	Methodology	Untreated GTR	GTR-MW	GTR+0.25B-MW	GTR+0.5B-MW	GTR+1.0B-MW	GTR+2.5B-MW
Si	WD-XRF	1.72	1.88	2.66	2.66	2.55	2.90
S		1.91	1.33	1.97	1.86	1.91	2.16
Zn		2.33	1.12	1.55	1.58	1.65	1.68
Ca		0.38	0.40	0.62	0.58	0.61	0.62
Al		0.07	0.08	0.1	0.12	0.12	0.14
Mg		0.06	0.06	0.07	0.08	0.06	0.09
Fe		0.14	0.04	0.05	0.05	0.05	0.07

**Table 5:** Volatile organic compounds determined using a SHS-GC-MS method.

Compound (mg/kg of Sample)	Untreated GTR	GTR-MW	GTR+0.25B-MW	GTR+0.5B-MW	GTR+1.0B-MW	GTR+2.5B-MW
Acetone	2.2	2.1	2.3	2.5	2.3	1.5
Methacrolein	0.9	0.5	0.7	0.6	0.7	0.5
2-methylfuran	1.3	0.6	0.9	0.8	0.9	0.6
Methyl vinyl ketone	1.6	0.9	1.3	1.0	1.3	0.9
Methyl isobutyl ketone	8.3	3.9	5.9	5.3	5.9	4.4
Cyclohexanone	2.4	1.6	2.2	1.9	2.2	1.8
Benzothiazole	6.5	6.9	7.9	7.6	7.9	7.6
Total content	23.2	16.5	21.2	19.7	21.2	17.3

**Table 6:** Thermal decomposition characteristics of reclaimed GTR estimated from TGA data.

Sample	T_−2%_ (°C)	T_−5%_ (°C)	T_−10%_ (°C)	T_−50%_ (°C)	T_max1_ (°C)	T_max2_ (°C)	Char Residues at 750 °C (%)
untreated GTR	258.5	304.3	341.3	448.6	384.8	434.4	40.0
GTR-MW	255.8	310.8	352.1	451.9	387.4	447.7	35.5
GTR+0.25B-MW	250.5	307.0	351.2	452.4	387.5	452.0	35.6
GTR+0.5B-MW	253.6	309.8	352.6	454.3	387.8	452.3	36.8
GTR+1.0B-MW	254.6	303.8	343.5	449.0	387.4	443.6	36.6
GTR+2.5B-MW	254.7	310.6	353.1	452.0	387.5	451.8	34.8

**Table 7:** Curing characteristics of tested samples.

Curing Characteristics at 150 °C	GTR-MW	GTR+0.25B-MW	GTR+0.5B-MW	GTR+1.0B-MW	GTR+2.5B-MW
M*_L_* (dNm)	16.9	17.9	29.5	28.1	24.4
M*_H_* (dNm)	40.9	40.4	51.0	48.9	46.0
ΔM (M*_H_*-M*_L_*) (dNm)	24	22.5	21.5	20.8	21.6
*t*_2_ (min)	2.5	2.6	2.5	2.5	2.5
*t*_90_ (min)	11.6	11.2	11.5	11.6	12.3
CRI (min^–1^)	10.9	11.6	11.1	11.0	10.2
R_300_ (%)	0.3	0.1	0.1	0.3	0.5

**Table 8:** Mechanical properties of tested samples.

Tensile Properties	GTR-MW	GTR+0.25B-MW	GTR+0.5B-MW	GTR+1.0B-MW	GTR+2.5B-MW
TS_b_ (MPa)	5.3 ± 0.7	5.9 ± 0.2	5.2 ± 0.8	6.1 ± 0.7	5.9 ± 0.7
E_b_ (%)	126 ± 15	139 ± 1	109 ± 5	138 ± 12	134 ± 13
M_100_ (MPa)	4.0	4.0	4.7	4.1	4.1
H (°Sh A)	64	63	67	67	65

**Table 9:** Comparison of tensile properties of reclaimed GTR prepared by different methods.

Reclaiming Method	GTR (mm)	Tensile Properties of Reclaimed GTR		Ref.
		TS_b_ (MPa)	E_b_ (%)	
Bitumen plasticization/microwave treatment	0.50	5.2–6.1	109–139	This study
Bitumen plasticization at ambient temperature	0.80	3.3–5.5	154–194	[49]
Microbial desulfurization	0.05	3.3	191	[50]
Shearing in pan mill reactor	0.25	4.2–8.4	109–202	[51]
Grinding, ultrasonically treated, ozone/ultrasonically treated	0.50	3.2–5.1	135–160	[52]
Thermo-mechanical in counter- and co-rotating twin screw extruder	1.50	3.3–6.5	114–180	[53]
